# Treating chronic atrophic gastritis: identifying sub-population based on real-world TCM electronic medical records

**DOI:** 10.3389/fphar.2024.1444733

**Published:** 2024-08-07

**Authors:** Yu-man Wang, Jian-hui Sun, Run-xue Sun, Xiao-yu Liu, Jing-fan Li, Run-ze Li, Yan-ru Du, Xue-zhong Zhou

**Affiliations:** ^1^ Graduate School of Hebei University of Traditional Chinese Medicine, Hebei, China; ^2^ Hebei Hospital of Traditional Chinese Medicine, Hebei, China; ^3^ Hebei Key Laboratory of Turbidity and Toxicology, Hebei, China; ^4^ Hebei Provincial Key Laboratory of Integrated Traditional and Western Medicine Research on Gastroenterology, Hebei, China; ^5^ School of Computer and Information Technology, Beijing Jiaotong University, Beijing, China

**Keywords:** real world, electronic medical record of Chinese medicine, chronic atrophic gastritis, population division research, data curation

## Abstract

**Background and Objective:**

Chronic atrophic gastritis (CAG) is a complex chronic disease caused by multiple factors that frequently occurs disease in the clinic. The worldwide prevalence of CAG is high. Interestingly, clinical CAG patients often present with a variety of symptom phenotypes, which makes it more difficult for clinicians to treat. Therefore, there is an urgent need to improve our understanding of the complexity of the clinical CAG population, obtain more accurate disease subtypes, and explore the relationship between clinical symptoms and medication. Therefore, based on the integrated platform of complex networks and clinical research, we classified the collected patients with CAG according to their different clinical characteristics and conducted correlation analysis on the classification results to identify more accurate disease subtypes to aid in personalized clinical treatment.

**Method:**

Traditional Chinese medicine (TCM) offers an empirical understanding of the clinical subtypes of complicated disorders since TCM therapy is tailored to the patient’s symptom profile. We gathered 6,253 TCM clinical electronic medical records (EMRs) from CAG patients and manually annotated, extracted, and preprocessed the data. A shared symptom-patient similarity network (PSN) was created. CAG patient subgroups were established, and their clinical features were determined through enrichment analysis employing community identification methods. Different clinical features of relevant subgroups were correlated based on effectiveness to identify symptom–botanical botanical drugs correspondence. Moreover, network pharmacology was employed to identify possible biological relationships between screened symptoms and medications and to identify various clinical and molecular aspects of the key subtypes using Gene Ontology (GO) and Kyoto Encyclopedia of Genes and Genomes (KEGG) pathway enrichment analysis.

**Results:**

5,132 patients were included in the study: 2,699 males (52.60%) and 2,433 females (47.41%). The population was divided into 176 modules. We selected the first 3 modules (M29, M3, and M0) to illustrate the characteristic phenotypes and genotypes of CAG disease subtypes. The M29 subgroup was characterized by gastric fullness disease and internal syndrome of turbidity and poison. The M3 subgroup was characterized by epigastric pain and disharmony between the liver and stomach. The M0 subgroup was characterized by epigastric pain and dampness-heat syndrome. In symptom analysis, The top symptoms for symptom improvement in all three subgroups were stomach pain, bloating, insomnia, poor appetite, and heartburn. However, the three groups were different. The M29 subgroup was more likely to have stomach distention, anorexia, and palpitations. *Citrus medica*, *Solanum nigrum*, Jiangcan, Shan ci mushrooms, and Dillon were the most popular botanical drugs. The M3 subgroup has a higher incidence of yellow urine, a bitter tongue, and stomachaches. *Smilax glabra*, *Cyperus rotundus*, *Angelica sinensis*, *Conioselinum anthriscoides*, and *Paeonia lactiflora* were the botanical drugs used. Vomiting, nausea, stomach pain, and appetite loss are common in the M0 subgroup. The primary medications are *Scutellaria baicalensis*, *Smilax glabra*, *Picrorhiza kurroa*, *Lilium lancifolium*, and *Artemisia scoparia*. Through GO and KEGG pathway analysis, We found that in the M29 subgroup, *Citrus medica*, *Solanum nigrum*, Jiangcan, Shan ci mushrooms, and Dillon may exert their therapeutic effects on the symptoms of gastric distension, anorexia, and palpitations by modulating apoptosis and NF-κB signaling pathways. In the M3 subgroup, *Smilax glabra*, *Cyperus rotundus*, *Angelica sinensis*, *Conioselinum anthriscoides*, and *Paeonia lactiflora* may be treated by NF-κB and JAK-STAT signaling pathway for the treatment of stomach pain, bitter mouth, and yellow urine. In the M0 subgroup, *Scutellaria baicalensis*, *Smilax glabra*, *Picrorhiza kurroa*, *Lilium lancifolium*, and *Artemisia scoparia* may exert their therapeutic effects on poor appetite, stomach pain, vomiting, and nausea through the PI3K-Akt signaling pathway.

**Conclusion:**

Based on PSN identification and community detection analysis, CAG population division can provide useful recommendations for clinical CAG treatment. This method is useful for CAG illness classification and genotyping investigations and can be used for other complicated chronic diseases.

## Graphical Abstract

**Figure:**
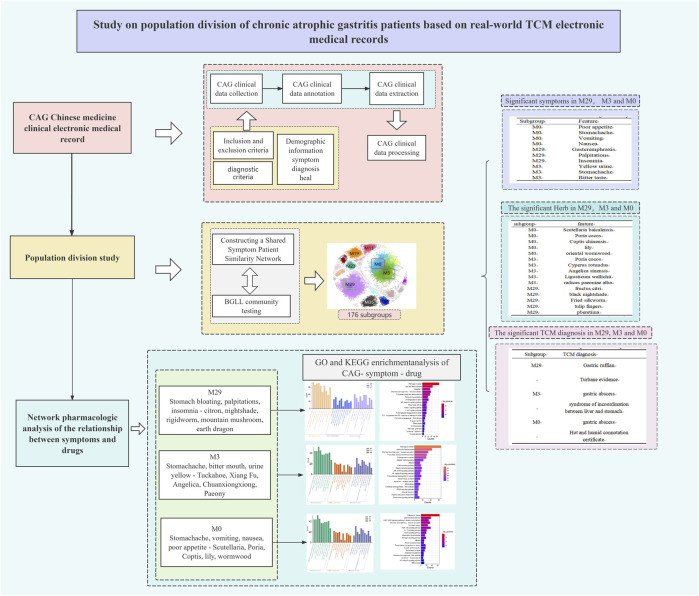
Technology roadmap

## 1 Background

Chronic atrophic gastritis (CAG) is one of the most common digestive system illnesses and is a significant risk factor for gastric cancer (Li et al., 2018).

A multifaceted cause of chronic disease, CAG has become an increasingly common health problem worldwide (Lahner et al., 2019; Shah et al., 2021). Furthermore, the detection and treatment of CAG have gradually increased the burden on humans. Atrophic gastritis is characterized by the loss of stomach glands with or without metaplasia, resulting from persistent inflammation caused by *Helicobacter pylori* or autoimmune response. Regardless of the cause, histology should corroborate the diagnosis of atrophic gastritis (Massironi et al., 2019; Zheng et al., 2023).

CAG is increasingly recognized as a highly diverse disease with a variety of clinical presentations (Pimentel-Nunes et al., 2019; Lahner et al., 2020). As a result, accurately identifying subgroups of CAG patients is critical for preventing the development of precancerous GC, determining customized therapeutic responses, and improving patient outcomes.

Precision medicine has been recognized as a novel medical tool for refining disease classification and increasing medical capacity (Zhou et al., 2018; Sisodiya, 2021). The division of a particular illness population is also a step towards precision medicine. The classification of diseases into subtypes based on distinct conditions aids in understanding illness causes and determining the clinical applicability of treatments (Yang et al., 2021). Currently, subtypes of cancer are primarily classified based on their molecular characteristics. However, morphologically identical tumours can exhibit diverse clinical characteristics and respond differently to treatment. The classification of subtypes can not only improve the creation of treatment strategies for the disease but also help to improve patient prognosis (Collisson et al., 2019). Traditional Chinese medicine (TCM) is a type of individualized medicine (Zhou et al., 2014; Xiang et al., 2019).

A comprehensive analysis of the results of TCM’s four diagnosis methods (looking, smelling, asking, and cutting) can be used to determine the disease’s symptom phenotype, divide the disease into different subtypes based on phenotypic characteristics, and prescribe individualized treatment plans for patients based on the characteristics of the different subtypes. This approach provides novel insights into illness status based on patient symptom features and herbal prescriptions.

This study gathered substantial real-world TCM clinical data on CAG patients to construct a shared symptoms patient similarity network (PSN). CAG patient subgroups were identified, and enrichment analysis was performed using community detection methods to determine the phenotypic characteristics of the distinct subtypes, which could aid in identifying clinical subgroups with clinical and biological importance. To investigate symptom-botanical drugs associations, the phenotypic characteristics of the major clinical subgroups were studied in terms of clinical efficacy. Enrichment analysis was utilized to discover clinical features as well as key biological pathways in the CAG patient population. Our findings are intended to serve as a benchmark for continuously optimizing individualized treatment options for CAG patients by improving personalized therapy and uncovering underlying causes.

## 2 Materials and methods

### 2.1 Data situation

#### 2.1.1 Data sources and preprocessing

From 2013 to 2023, the EMR database of the Hebei Hospital of Traditional Chinese Medicine collected data on 6253 CAG inpatients, including all information obtained during hospitalization, such as demographics, symptoms, diagnosis, and treatment. We employed clinical information extraction methods because the majority of the data were free text and could not be directly analysed (Shu et al., 2019). Biomedical factors (for instance, TCM diagnosis, symptoms, and prescriptions) were successfully derived from these records. To standardize the descriptions of the diverse clinical terminologies, we referred to the International Classification of Diseases 10th Edition (ICD-11) (The Lancet 2019) the Pharmacy Handbook of the People’s Republic of China (Revision 2020) (ChP 2020), and the botanical drugs Bank Online (Wishart et al., 2018). Manual inspection and standardization were undertaken for the terms “disease diagnosis,” “herbal,” and “botanical drugs,” respectively.

We standardized the nomenclature for symptoms, herbs, and diagnosis using the Medical Integrator Studio, a platform established by a TCM clinical data warehouse (Shu et al., 2019). An extraction conversion loading (ETL) tool was also used. Diseases with detailed ICD-11codes were grouped according to the higher-level codes. For example, the ICD-11 codes I50.903 and I50.905 were combined into the ICD-11 code I50.9.

#### 2.1.2 Diagnostic criteria

It is consistent with the Diagnosis and Treatment Plan of Integrated Chinese and Western Medicine for Chronic Gastritis (2003) and the Consensus Opinion on the diagnosis and Treatment of Integrated Chinese and Western Medicine for Chronic Atrophic Gastritis (2017).

Western Medicine disease name code: Refer to the International Classification of Diseases 11th Revision (ICD-11), which is consistent with the disease code used in the hospital medical record information system.

#### 2.1.3 Inclusion and exclusion criteria

##### 2.1.3.1 Inclusion criteria

① Patients who met the above diagnostic criteria and were first diagnosed as “chronic atrophic gastritis” according to discharge diagnosis, that is, patients with ICD-10 code “K29.400”; ② The time of admission was from 2013 to 2023; ③ The patient’s basic information, examination information and discharge record (discharge diagnosis) information are complete.

##### 2.1.3.2 Exclusion criteria

1) Patients with missing necessary information: patients with missing patient ID, diagnostic information, and discharge record information; 2) Only the most complete hospitalization information is retained when the same patient is hospitalized multiple times.

### 2.2 Network pharmacology methods

The SwisTargetPrediction platform (http://www.swistargetprediction.ch/) and the botanical drugsBank database (https://go.botanicaldrugsbank.com/) were used to retrieve the active metabolites of relevant botanical drugs, predict the botanical drugs target, and create a botanical drugs target database after sorting, merging, and removing weight. After utilizing the GeneCards database (http://www.genecards.org/) to determine distinct subgroups according to “chronic atrophic gastritis” and related symptoms, the jvenn platform (https://jvenn.toulouse.inrae.fr/app/example.html) was used to generate a Venn diagram to determine intersection targets. Phenotype-genotype and herbal-target associations (Wu et al., 2019) were retrieved from the SymMap database.

Afterwards, the intersection targets were imported into the STRING database (https://cn.string-db.org/cgi/input) to construct a PPI network. Next, different subgroups of CAG botanical drugs therapy targets were identified via the Metascape platform, and *Homo sapiens* was selected as the species for Gene Ontology (GO) and Kyoto Encyclopedia of Genes and Genomes (KEGG) functional and pathway enrichment analyses.

### 2.3 Construction of the patient similarity network

We used the Jaccard coefficient (Niwattanakul et al., 2013) to construct the PSN. Patient similarity was calculated based on the patient symptom phenotype, and a patient similarity network was constructed, defined as Jaccardsim (A, B) = P (AB)/P (AB); this calculation shows that if two patients have more crossover in their symptom phenotypes, they will have greater symptom similarity. Specifically, if two patients have the same symptom phenotype, they have a symptom similarity of 1. Since symptoms and current history of the complaint contribute differently to diagnosis and treatment, we combined three steps to construct the PSN. First, we calculated the patient similarity based on the main symptoms (Jaccard similarity) to form the PSN of the main symptoms. We then filtered the edges by a weight of 0.8 to keep only the patients associated with almost the same predominant symptoms. Second, patient similarity was calculated from the general symptoms (phenotype in the current history) and used to construct the PSN for general symptoms. Finally, we obtained the overlap of the two PSNs where the patient similarity of the latter served as a weight link.

### 2.4 Community testing was used to identify patient subgroups

We applied two efficient community detection algorithms, namely, BigClam (2013) and BGLL (Blondel et al., 2008). Patient subgroups or disease comorbid subnetworks were obtained from the entire network. For LDCN, CAG can be visually identified simultaneously, and an overlapping subnetwork structure needs to be identified. Therefore, we used BigClam, a high-performance overlap community detection algorithm based on nonnegative matrix decomposition (NMF). BigClam can detect densely overlapping, hierarchically nested communities in large-scale networks. This algorithm differs from other algorithms because it detects overlapping communities by using maximum likelihood estimates instead of modularity and matrix eigenvalues. We used the Stanford Network Analysis Project (SNAP) (Leskovec and Sosič, 2016) as the platform for the BigClam algorithm. For networks with similar symptoms, all nodes are patients, which we wanted to divide into subgroups with clearer boundaries, so a nonoverlapping community detection method is more applicable. BGLL is an iterative algorithm based on modularity measures that achieves the maximum gain of modularity through continuous division, combining local optimization and multilevel clustering, and it has linear complexity on typical and sparse data.

### 2.5 Phenotype enrichment analysis

Relative risk (RR) is a classical statistical method. RR is the ratio of the probability of an event occurring in the exposed group (Ouimet et al., 2010a) to that in the non-exposed group. In this study, we used RR to assess the specificity of symptoms, herbs and disease in each patient module. We treated the patients in the specific module as the exposed group, the remaining patients as the non-exposed group, and the symptoms as the event. Therefore, RR is defined as RR= (C ij/Ci)/((Cj C ij)/(NCi)), where the number of patients in the Ci module (patient subgroup) is i, Cj is the number of patients with symptoms j, C ij is the number of patients in module i with symptom j, and N is the total number of patients in this study. If RR > 1, the distribution of symptom j in module i is greater than the overall distribution. Similar approaches were also used to identify important herbs and diseases in specific patient subgroups. In addition, true significant symptoms, herbs, or diseases were filtered out using the chi-square test (*p*-value < 0.05), a statistical hypothesis test, the results of which were assessed with reference to card variance (Pirhaji et al., 2008a).

### 2.6 Gene ontology (GO) and KEGG pathway enrichment analysis

GO and KEGG pathway enrichment analyses help to trace both DNA correlation and protein-related issues. They provide considerable power for the discovery of the biological functions of genes and proteins (Cieslak et al., 2020). The Gene Ontology (GO) project is a comprehensive resource for functional genomics. This project created annotations supported by evidence to describe the biological roles of individual genomic products (e.g., genes, proteins, ncRNA complexes) (Pirhaji et al., 2008). Pathway analysis provides considerable power to discover the biological functions of genes and proteins. The KEGG pathway database is the main database of the Kyoto Encyclopedia of Genes and Genomes (KEGG) and consists of manually drawn reference pathway maps and organism-specific pathway maps (Ouimet et al., 2010). We obtained the relevant GO and KEGG pathways using the Annotation, Visual and Integrated Discovery database (Metascape), a web-based online bioinformatics resource designed to provide tools for the functional interpretation of a large number of genes/proteins (Chen et al., 2017).

## 3 Results

### 3.1 Basic statistical characteristics of 6,253 hospitalized CAG patients

According to the discharge data of 6,253 patients, 66.1% of the hospitalized CAG patients improved, and 32.9% left the hospital after receiving medical advice (the condition was relatively better, so the patients were discharged). Only three of these patients died during the course of the disease (Table 1), and these patients were over 80 years old. Through data annotation and screening of 6,253 patients, a total of 5,132 patients were included in the analysis, including 2,699 (52.60%) males and 2,433 (47.41%) females. The majority of CAG patients (47.75%) were between 60 and 79 years old. Moreover, the basic information of CAG patients between 40 and 59 years (43.78% of patients) of age was comparable (Table 2). It may be speculated that most CAG patients are middle-aged or elderly. However, the possibility of CAG in adolescent patients is not excluded.

**TABLE 1:** 6,253 patients.

Follow-up	Count	Proportion (%)
Improvement	3,375	65.8
Discharge from the hospital	1,746	34.0
Death	3	<0.1
Receipt of medical advice from the hospital	3	<0.1
Other	2	<0.1
Left hospital against doctor’s advice	1	<0.1
Recovery	1	<0.1
Automatic discharge	1	<0.1

**TABLE 2:** Characteristics of 5113 CAG inpatients.

Characteristics		n (%)/(mean ± SD)
Sex	Male	2,699 (52.60)
	Female	2,433 (47.41)
Age		59.27 ± 11.44
Age group	<20	5 (0.1)
	20–39	257 (5.03)
	40–59	2,247 (43.78)
	60–79	2,450 (47.74)
	≥80	172 (3.36)
Nationality	Han nationality Minority nationality	5,078 (99) 54 (1)

### 3.2 CAG patient similarity network

**FIGURE 1:**
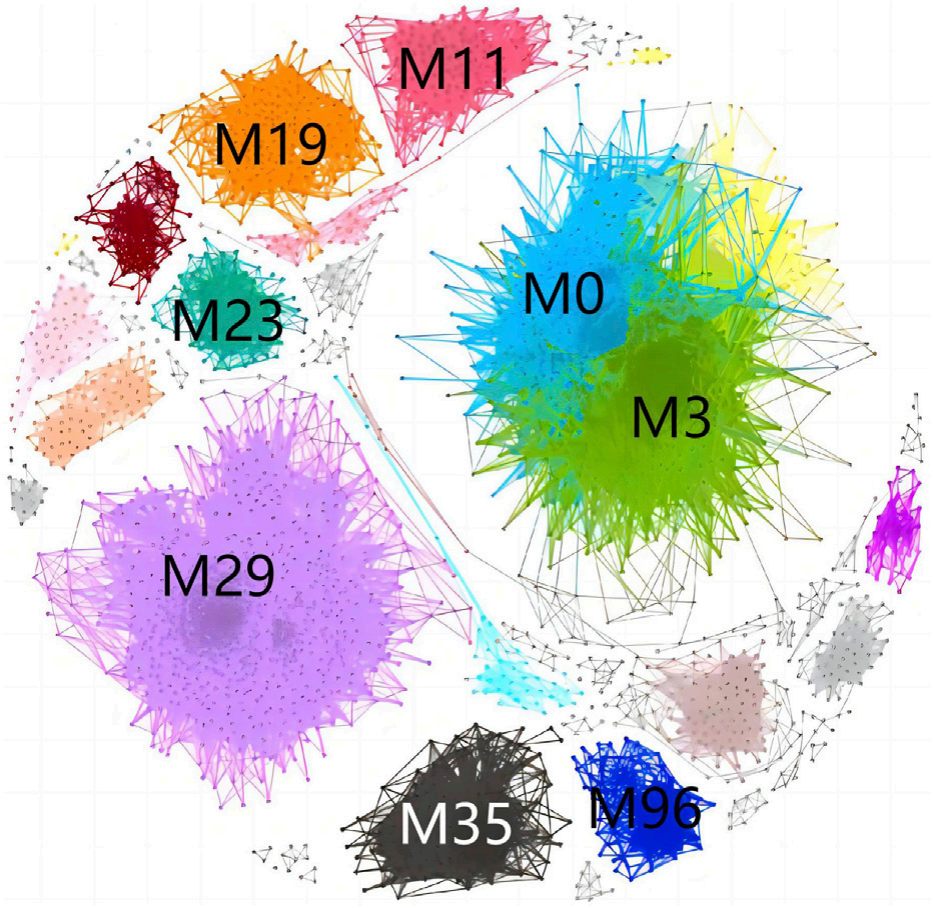
Community structure of the patient similarity network. Different colours represent different modules.

**FIGURE 2:**
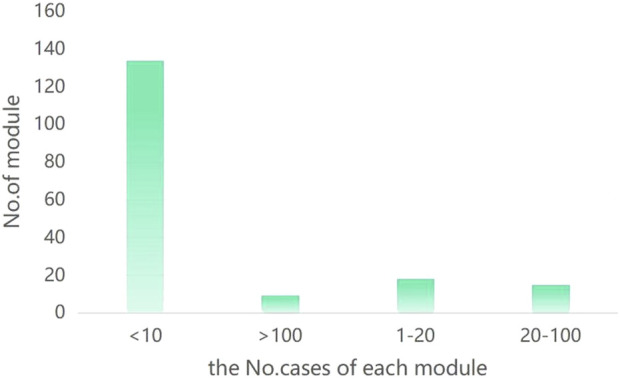
Number of patients in each module.

To identify the disease subtypes of CAG patients, we first calculated the extent to which symptom phenotypes were shared between patient pairs and constructed a PSN with 4,418 nodes (patients) and 190,385 connected edges. Next, we applied a high-performance community detection method (see Materials and Methods) to explore the subgroups (corresponding to patients with disease subtypes). We obtained 176 modules, with the number of patients per module ranging from 2 to 860 (Figure 1). These modules represent a subgroup of CAG patients, with eight modules (29, 3, 0, 19, 11, 35, 23, 96) with 100 or more cases representing 67% of the entire cohort and 137 modules with 10 or fewer cases representing approximately 9.78%. The number of cases in the eight largest modules was as follows: M29 = 860 (19.5%), M3 = 818 (18.5%), M0 = 419 (9.5%), M19 = 249 (5.6%), M11 = 199 (4.5%), M35 = 152 (3.4%), M23 = 120 (2.7%), and M96 = 113 (2.6%) (Figure 2). Overall, these eight modules accurately reflect the clinical characteristics and general characteristics of CAG patients. The medium-sized modules (67, 52, 45, 1) reflect the individual characteristics of CAG patients. Small modules may represent a minority group. Finally, considering the precision treatment of CAG patients, we selected the 3 largest modules (M29, M3, M0), accounting for 47.5% of the entire cohort, to illustrate the characteristic phenotype and genotype of the CAG disease subtype.

### 3.3 Significant clinical phenotypes in the CAG subgroup

We then screened for different phenotypic features of the selected modules using RR and chi-square tests (RR ≥ 1, *p* < 0.05). To determine the significant symptoms of the different modules, TCM syndrome, tongue, and pulse characteristics were evaluated.

First, for the significant clinical phenotype of each subgroup, the top-ranked features were selected according to the RR ranking. First, the symptoms of the three modules were studied by RR ranking. Patients in the M0 module are more likely to exhibit symptoms such as poor appetite, stomachache, vomiting, fatigue, nausea, and mouth adhesion. Patients in the M29 module are more prone to stomach distension, insomnia and palpitations. Patients in the M3 module are more prone to stomach pain, bitter mouth and yellow urine. Interestingly, the lingual veins of patients in M29 are purple, dark, and thin; the veins of patients in M3 are a dark red and smooth with yellow and greasy moss-like appearance; and the veins of patients in M0 are red and yellow, thin and smooth (Tables 3–5). Interestingly, in terms of TCM diagnosis and syndrome type, the patients in the M29 module are characterized by gastric Ruffian disease and turbidity poisoning. The patients in the M3 module are characterized by epigastric pain and liver and gastric incompatibility. The patients in the M0 module are characterized by epigastric pain and internal evidence of heat and dampness (Table 6). This finding is consistent with previous symptoms, tongue veins and other clinical symptoms.

**TABLE 3:** Significant symptoms in M29, M3 and M0.

Subgroup	Feature	chiq_p	RR
M0	Poor appetite	0.000536052	1.157249561
M0	Stomachache	5.7221E-59	1.779952943
M0	Vomiting	1.80144E-05	1.830385458
M0	Nausea	0.001071615	1.421817275
M29	Gasteremphraxis	2.96779E-73	1.486584353
M29	Palpitations	0.000116052	4.137209302
M29	Insomnia	0.010708801	4.137209302
M3	Yellow urine	1.61319E-07	1.747906154
M3	Stomachache	9.4091E-127	1.951496274
M3	Bitter taste	0.009596049	1.130851593

**TABLE 4:** Significant tongue features in M29, M3 and M0.

Subgroup	Feature	chiq_p	RR
M0	Thin moss	2.5024E-08	1.460670333
M0	Red tongue	3.2305E-225	5.534004533
M0	Greasy fur	7.7694E-05	1.107231526
M0	Yellow moss	1.01875E-06	1.084441699
M3	Greasy fur	0.028959939	1.044881381
M3	Dark red tongue	1.34934E-34	1.357292771
M3	Yellow moss	0.009131126	1.034161702
M3	Greasy fur	0.028959939	1.044881381
M29	Dark purple tongue	0.024758658	1.510980789

**TABLE 5:** Significant pulse patterns in M29, M3 and M0.

Subgroup	Feature	chiq_p	RR
M0	Thin pulse	7.73728E-08	1.582450196
M0	Slippery pulse	0.005758758	1.057385832
M0	Thready pulse	3.95726E-11	1.134950254
M3	Thready pulse	1.2622E-28	1.173206935
M3	Slippery pulse	1.16501E-32	1.187797111
M29	Thin pulse	2.33312E-09	1.495904725
M29	Thready pulse	2.40389E-27	1.165878014

**TABLE 6:** The significant TCM diagnosis in M29, M3 and M0.

Subgroup	TCM diagnosis	chiq_p	RR
M29	Gastric ruffian	<0.01	4.038592509
	Turbane evidence	9.5506E-121	1.727184466
M3	Gastric abscess	1.9317E-208	2.482758621
	Syndrome of incoordination between liver and stomach	1.3135E-113	1.712654615
M0	Gastric abscess	5.74981E-97	2.162790698
	Hot and humid connotation certificate	3.23E-53	1.598960416

### 3.4 Significant symptom-botanical drugs associations in the clinical phenotype subgroups in CAG

Next, we compared the patients’ symptoms on admission with those after discharge (Supplementary Table S1). The aim of this analysis was to determine the link between specific symptoms and medication. Because more distinguishing subgroups were identified, fewer patients were included in the subsequent subgroups. Finally, the three (subgroups 29, 3, and 0) with the greatest similarity coefficients were selected, and the community analysis results were analysed (Supplementary Table S2). The phenotypes of clinical symptoms versus medication phenotypes in the three subgroups were analysed using python.

**FIGURE 3:**
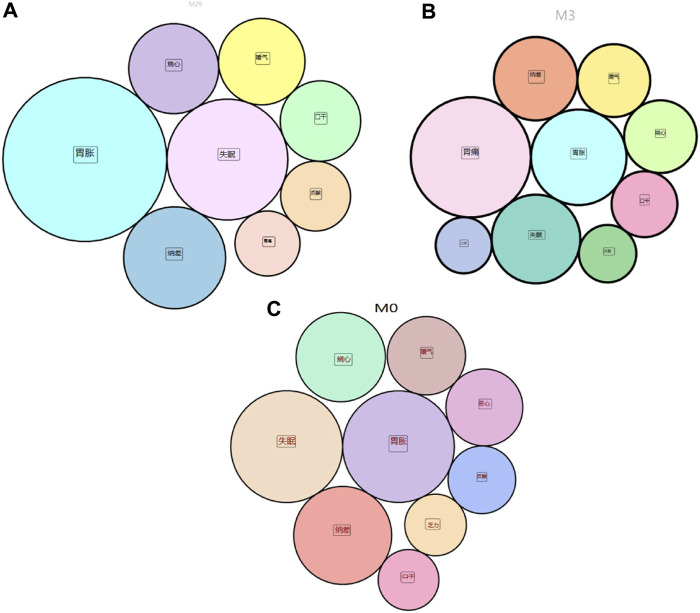
**(A)** disappearance of symptoms in the M29 subgroup; **(B)** disappearance in the M3 subgroup; **(C)** disappearance in the M0 subgroup.

In the M29 subgroup, the overall symptoms improved at discharge, and the specific symptoms of gastric distension, insomnia, poor appetite, heartburn, belching, dry mouth, acid reflux, and stomach pain all disappeared (Figure 3A; Supplementary Table S3). The results from python for symptom efficacy were consistent with the previous screening of different phenotypic characteristics of the modules using RR and chi-square tests (RR ≥ 1, *p* < 0.05). Next, we analysed the medication status of patients in the M29 subgroup and found that citron, dragon sunflower, fried silkworm, mountain mushroom and ground dragon were critical traditional Chinese medicines in the M29 module (Table 7). The M29 subgroup of patients showed better medication efficacy, and we speculated that the disappearance of symptoms may be related to the application of these Chinese medicines. In the M3 subgroup, the overall symptoms improved at discharge, and the specific symptoms of stomach pain, stomach distension, insomnia, poor appetite, belching, heartburn, dry mouth, acid reflux, and bitter mouth all disappeared (Figure 3B; Supplementary Table S4). The results from python for symptom efficacy were consistent with the previous screening of different phenotypic characteristics of the modules using RR and chi-square tests (RR ≥ 1, *p* < 0.05). Next, we analysed the medications used by patients in the M3 subgroup and found that Poria, Vinegar, Angelica, Chuanxiong, and white peony roots were critical Chinese medicines in the M3 module (Table 7). The patients in the M3 subgroup showed better efficacy, and we speculated that the disappearance of symptoms may be related to the application of these Chinese medicine.

**TABLE 7:** The significant Herb in M29, M3 and M0.

Subgroup	Feature	chiq_p	rr
M0	Scutellaria baicalensis	0.005231395	1.117733892
M0	Poria cocos	3.72721E-05	1.174208528
M0	Coptis chinensis	0.000407442	1.151168479
M0	Lily	0.002969888	1.16017854
M0	Oriental wormwood	0.015986414	1.130789665
M3	Poria cocos	4.01248E-10	1.194553119
M3	Cyperus rotundus	0.041862696	1.069116633
M3	Angelica sinensis	7.39513E-05	1.136624869
M3	Ligusticum wallichii	0.000461076	1.180341211
M3	Radices paeoniae alba	7.79748E-12	1.256531134
M29	Fructus citri	2.71549E-10	1.882503604
M29	Black nightshade	0.044947528	4.851655629
M29	Fried silkworm	3.30196E-05	1.600546187
M29	Tulip fingers	0.020931945	1.852450331
M29	Pberetima	0.019449927	1.530137545

In the M0 subgroup, the overall symptoms, including stomachache, insomnia, poor appetite, heartburn, belching, nausea, acid reflux, and fatigue, improved at discharge (Figure 3C; Supplementary Table S5). The results obtained by python were relatively consistent with the previous screening of the different phenotypic characteristics of the modules using RR and chi-square tests (RR ≥ 1, *p* < 0.05). Next, we analysed the medications used in patients in the M0 subgroup and found that baicalensis, Poria, Coptis, lily, and wormwood were critical Chinese medicines in the M0 module (Table 7). The patients in the M0 subgroup showed better efficacy, and we speculated that the disappearance of symptoms may be related to the application of these Chinese medicine.

We analysed the symptoms of the M29, M3 and M0 subgroups. The symptoms with the greatest improvement in the three subgroups were stomach pain, stomach distension, insomnia, poor appetite, and heartburn. However, the rankings of symptoms according to improvement are different. We speculate that this may be because patients with chronic atrophic gastritis mainly show these symptoms clinically and that the characteristic botanical drugs used in each subgroup have some effect on the universal symptomatic treatment of CAG. In addition, botanical drugs have a certain bias in the treatment of symptoms. Interestingly, in the previous analysis, we identified the M29, M3 and M0 subgroups. What is the association between botanical drugs and symptoms? Therefore, we combined the five universal symptoms with the respective characteristic symptoms of M29, M3 and M0 to determine molecular associations with the botanical drugs analysed in each subgroup and then performed GO and KEGG analyses. The relevant targets and mechanisms of different botanical drugs for treating symptoms in different subgroups of patients can be explored through molecular studies.

## 4 Network pharmacology analysis of molecular associations in the M29, M3 and M0 subgroups

We selected M29 (symptoms: stomach pain, stomach distension, insomnia, poor appetite, heartburn, and palpitations; botanical drugs: citron, sunflower, fried silkworm, mushroom, and dragon), M3 (symptoms: stomachache, stomach distension, insomnia, poor appetite, belching, bitter mouth, yellow urine; botanical drugs: poria cocos, vinegar, angelica, xiong, white peony root), and M0 (symptoms: stomach pain, stomach distension, insomnia, poor appetite, heartburn, nausea, vomiting; botanical drugs: Scutellaria baicalensis, Poria cocos, Coptis chinensis, lily, wormwood) for related pharmacological studies. Network pharmacology methods were applied to explore the shared molecular associations between important TCM-symptom-CAG connections in M29, M3 and M0. First, we identified the chemical components and associated genes associated with each of the important Chinese medicines, symptoms, and symptoms in M29, M3, and M0 from an external database (see Materials and Methods). The target information for CAG was collected.

Then, we imported the information into the String database to obtain the PPI core target network map. Finally, the core targets from the final screen were imported into the Metascape database for GO functional enrichment analysis and KEGG signalling pathway annotation analysis. The WeChat Letter website was used to visualize the results.

**FIGURE 4:**
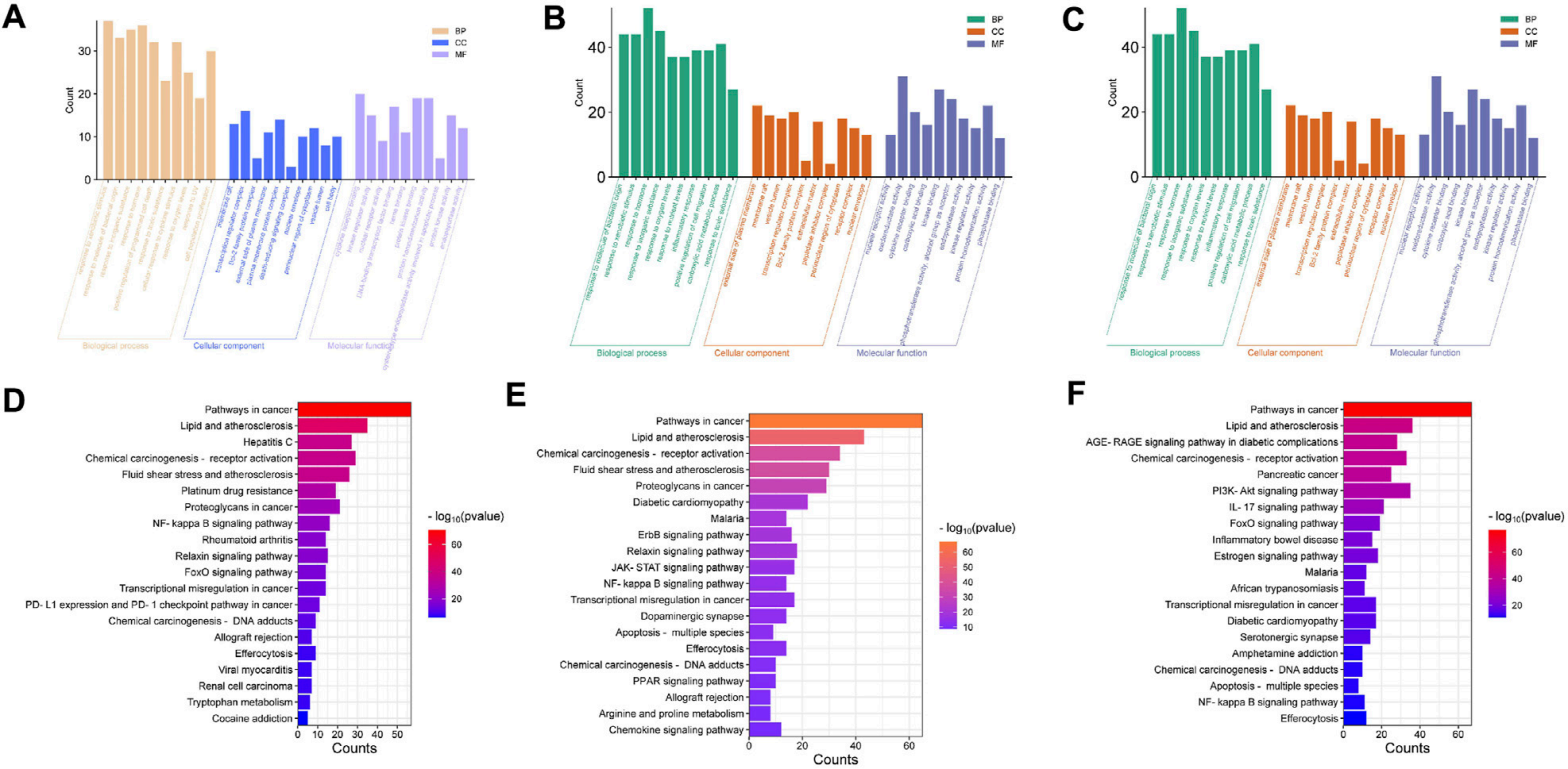
**(A)** GO enrichment analysis of M29 CAG-symptom-botanical botanical drugs associations. Three-in-one diagram of biological process, cellular component and molecular function, top 10 terms are shown; **(B)** GO enrichment analysis of M3 CAG-symptom-botanical botanical drugs associations. Three-in-one diagram of biological process, cellular component and molecular function, top 10 terms are shown; **(C)** GO enrichment analysis of M0 CAG- symptom-botanical botanical drugs associations. Three-in-one diagram of biological process, cellular component and molecular function, top 10 terms are shown; **(D)** Histogram of the top 20 KEGG pathways for M29 CAG-symptom-botanical drugs association. **(E)** Histogram of the top 20 KEGG pathways for the M3 CAG-symptom-botanical drugs association. **(F)** Histogram of the top 20 KEGG pathways for the M0 CAG-symptom-botanical drugs association. The entries represent the 1 * log10 (*p*-value) values.

The Metascape gene enrichment online analysis website was used to analyse 107 potential target genes of the M29 CAG-symptom-botanical botanical drugs subgroup, and GO biological process, GO cellular component and GO molecular function enrichment analyses were performed (Figure 4A). The potential target genes in the GO biological process category are mainly enriched in response to external stimuli, response to bacterial molecules, response to inorganic substances, response to hormones, positive regulation of programmed cell death, and cell response to cytokine stimulation; these findings may suggest that the botanical drugs used in the M29 subgroup affect diseases related to various responses. In addition, a total of 192 pathways were enriched according to KEGG enrichment analysis, of which the 20 most important pathways are shown in Figure 4D. Based on these results, we hypothesized that the therapeutic effect of the botanical drugs used in the M29 subgroup may treat CAG through pathways involved in cancer, the NF-kappa B signalling pathway, the relaxin signalling pathway, and the FoxO signalling pathway.

The 178 potential target genes in the M3 subgroup were also more involved in GO biological processes, which mainly included the response to molecules of bacterial origin and the inflammatory response. It is speculated that the botanical drugs used in the M3 subgroup may exert their therapeutic effect by intervening in the inflammatory response. The genes enriched in GO cellular component and GO molecular function terms were mainly correlated with the external side of the plasma membrane, membrane rafts and oxidoreductase activity (Figure 4B). It is speculated that the M3 subgroup may regulate disease by acting on the cell membrane and regulating oxidoreductase activity. In addition, 214 pathways were identified according to KEGG enrichment analysis, and the 20 most important pathways are shown in Figure 4E. Based on these results, we hypothesized that the therapeutic effect of the botanical drugs used in the M3 subgroup might involve a mechanism of action through the NF-kappa B signalling pathway and the JAK-STAT signalling pathway.

The 149 potential target genes in the M0 subgroup were also more involved in GO biological processes, which mainly included cellular response to lipids, cell population proliferation, and positive regulation of programmed cell death. GO cellular component and GO molecular function terms mainly included transcription regulator complex, membrane raft, cytokine receptor binding and transcription factor binding (Figure 4C). It is speculated that the botanical drugs used in the M0 subgroup may regulate disease by acting on the cell membrane and regulating the cytokine receptor binding and transcription factor binding processes. In addition, 204 pathways were identified according to KEGG enrichment analysis, and the 20 most important pathways are shown in Figure 4F. Based on these results, we hypothesized that the therapeutic effects of the botanical drugs used in the M0 subgroup may exert their mechanism of action through pathways involved in cancer, the PI3K-Akt signalling pathway, the IL-17 signalling pathway, and the FoxO signalling pathway.

## 5 Discussion

CAG, as a multifactor, potentially chronic disease, has become an increasingly common health problem worldwide (Li et al., 2024). However, in terms of treatment, no specific Western medicine has been identified, and therapeutic approaches mainly rely on symptomatic treatment (Yan-Rui et al., 2024a). TCM treatment for CAG involves classifying the disease type based on clinical characteristics, such as four sets of diagnostic information, according to the overall disease, syndrome differentiation and treatment. Next, the prescription botanical drugs used for treatment are determined (Chen et al., 2024a). TCM has good clinical efficacy in the treatment of CAG (Huang and Shao, 2024; Huang and Shao, 2024; Yan-Rui et al., 2024; Zhang et al., 2024). In addition, the treatment of CAG with traditional Chinese medicine is a similar process to that of the precision medicine currently advocated. Precision medicine has been recognized as a new medical method that can refine disease classification and improve medical capacity. Classification of the disease population is the prelude to precision medicine. Classification of the disease into subtypes according to different conditions helps to explore disease pathogenesis and determine the clinical operability of the treatment (Lehrich and Delgado, 2024).

At present, the clinical study of chronic atrophic gastritis is still focused on RCTs and studies with small sample sizes. One problem is that the small sample size does not fully represent the CAG population. With the development of informatization and data in the medical industry, electronic records of cases treated with TCM are becoming increasingly popular, improving the problems of insufficient sample size (Ma and Zhang, 2024). To better explore the effects of TCM treatment on CAG, the botanical drugs-evidence relationship was studied. This study included data from 6,253 CAG inpatients collected from the EMR database of Hebei Provincial Hospital from 2013 to 2023. We manually annotated, extracted, and preprocessed the collected EMR data of the CAG patients. Improving the related reports of TCM and syndromes leads to the mismatching of terms containing semantically similar but lexically different terms. We addressed the problems of missing data and semantic mismatch by building a shared symptom patient similarity network (PSN). The community analysis method distinguished the subgroups of patients with CAG. Through in-depth analysis of the botanical drugs-symptom relationships in the three most significant subgroups, the clinical characteristics and medication characteristics of the CAG patients were explored. We subsequently performed network pharmacology analysis of the botanical drugs symptoms obtained from the three subgroups individually and explored the possible molecular associations and therapeutic mechanisms between botanical drugs and symptoms through GO and KEGG pathway enrichment analysis.

The study revealed that stomachache, stomach distension, insomnia, poor tolerance, and heartburn symptom phenotypes were prevalent in the three subgroups. Each subgroup had its own characteristic symptom phenotype. This finding is consistent with the syndrome differentiation and classification of chronic atrophic gastritis in the consensus opinion of TCM diagnosis and treatment of chronic gastritis (2017) (Zhang et al., 2017). The M29 subgroup was more likely to have symptoms of gastric distension, anorexia, and palpitations. The tongue veins are characterized by purple, dark, thin veins. In traditional Chinese medicine, these symptoms are attributed to gastric ruffian disease and turbidity poison. Citron, nightshade, fried silkworm, mountain mushroom and ground dragon are the preferred medications in this group. Citron is a qi medicine, and modern pharmacology shows that citron has good antioxidative, anti-inflammatory, antiallergic, antibacterial, antitumour and other pharmacological effects. Citron is highly effective in treating diseases caused by poor ventilation (Yan et al., 2022). Citron is justified for treating symptoms in the M29 subgroup of CAG patients. Dragon sunflower and mountain kindness mushrooms can clear heat and detoxify the patient, promoting blood circulation and removing blood stasis. TCM states that “long disease can be treated from silt” or “long disease into the collateral.” CAG is a chronic disease, and in the M29 module, turbidity poison is an indicator of the late stage of CAG. During treatment, dragon sunflower and the shanici mushroom can be used to promote blood circulation and remove blood stasis (Wang et al., 2021; Li et al., 2024). The stiff silkworm and ground dragon play roles in promoting blood circulation and dredging collaterals in the treatment of CAG (Liang et al., 2024). Patients in the M3 module are more prone to stomach pain, bitter mouth and yellow urine. The tongue vein examination shows a dark red tongue, yellow and greasy moss, and smooth veins. In traditional Chinese medicine, epigastric pain is a liver and stomach incompatibility syndrome. Among the identified botanical drugs, Poria, incense, angelica, chuanxiong and white peony root were used in the M3 module for pain relief. Poria cocos strengthens the spleen and regulates qi, which regulates gastrointestinal symptoms and combats the qi machine (Jiang et al., 2021). Angelica Chuanxiong treats blood circulation qi, and white peony roots treat blood circulation, consistent with the “qi and blood origin, blood is the mother of qi.” The fragrance promotes qi circulation and reconciliation, resolves phlegm and relieves pain, which is consistent with the symptoms observed in the M3 module (Zhan et al., 2021). Patients in the M0 module are more likely to show symptoms of poor tolerance, stomach pain, vomiting and nausea. The tongue pulse is red, yellow, greasy, and smooth. The M0 module is characterized by epigastric pain and internal evidence of heat and dampness. *Scutellaria baicalensis*, *Poria cocos*, *Coptis chinensis*, lily and wormwood were used in this module. Patients in the M0 subgroup were advised to clear heat and dampness and relieve pain. The selected botanical drugs include baicalensis and Coptis affects yin to clear heat and dampness (Tian et al., 2016; Wang et al., 2017); *Poria cocos* manages spleen qi, which clears dampness and heat-injured yin; the Yin fluid in the human body is missing, and the lily can nourish yin and moisten dryness to supplement the Yin fluid in the body. This study used the similarity coefficient and community detection method and, according to the patient botanical drugs-symptom relationships, determined the tongue-pulse-symptoms-syndrome-botanical drugs relationships, which are similar to the traditional Chinese medicine dialectical classification. To a certain extent, the objectivity of traditional Chinese medicine syndrome can provide important guidance for the clinical use of traditional Chinese medicine. This method can be used for other complex diseases.

To explore the specific pathogenic mechanism underlying the associations between botanical drugs and symptoms, we used a network pharmacology method to analyse the molecular associations between botanical drugs. GO and KEGG pathway enrichment analyses were performed to identify different clinical and molecular features of important subtypes. TCM treatment of diseases operates on a deeper molecular level.

We speculate that the potential target genes in the M29 subgroup are affected mainly through the positive regulation of programmed cell death. Chen et al. (2024b) reported that ZJP treated CAG by inhibiting inflammation, inhibiting apoptosis through the PI3K/Akt signalling pathway and protecting the gastric mucosal barrier. Guo et al. (2024) found that XLHZ improved the pathological state and ultrastructure of the gastric mucosa by regulating the YY1/miR-320a/TFRC signalling pathway, inhibiting iron death and hindering the development of CAG. In addition, KEGG enrichment analysis revealed a possible association with the NF-kappa B signalling pathway and FoxO signalling pathway. Xie et al. (2024) found that WFC suppressed inflammation by inactivating the NF-κB pathway and preventing gastrointestinal metaplasia and dysplasia associated with inflammation. Lou et al. (2024) performed network pharmacology and *in vivo* experimental studies and revealed that the efficacy and mechanism of MLD treatment in CAG may involve the NF-κB signalling pathway. Tabassam et al. (2012), we found that FoxO1 and FoxO3a, as novel nuclear substrates of the *H. pylori*-induced PI3K/Akt cell survival signalling pathway, could partially control the production of interleukin 8, which exerts its anti-*H. pylori* effects and subsequently exerted their effects on the treatment of CAG.

We speculate that potential TCM treatment target genes in the M3 subgroup are associated with the inflammatory response. Chen et al. (2024) additionally, Qi-Zhi-Wei-Tong particles alleviate inflammatory responses in mice by changing the gut microbiota and bile acid metabolism to treat CAG. According to the KEGG enrichment analysis, the therapeutic effect of botanical drugs in the M3 subgroup may involve the NF-kappa B signalling pathway and the JAK-STAT signalling pathway. Buzzelli et al. (2019) reported that the cytokine IL-11 is specifically overexpressed in the stomach and can trigger spontaneous atrophic gastritis via the gp 130-JAK-STAT 3 signalling pathway.

The 149 potential target genes in the M0 subgroup were associated with cellular response to lipids, cell population proliferation, and positive regulation of programmed cell death. This finding is similar to that of the M29 subgroup, both of which are associated with cell death. KEGG enrichment analysis revealed that the M0 subgroup may be associated with PI3K-Akt signalling, IL-17 signalling, and FoxO signalling. Tong et al. (2021) reported that ZJP intervention significantly reduced the levels of G-17 and the inflammatory factors IL-8, TNF-α, IL-6 and IL-1β; inhibited the expression of TGF-β1, PI3K and its downstream signals p-Akt, p-mTOR, and P70S6K; and promoted the expression of PTEN, LC3-II and Beclin-1. Hayashi et al. (2012) reported that IL-17 could promote the colonization of *Helicobacter pylori* and promote the occurrence of an inflammatory response in chronic atrophic gastritis patients.

In this study, CAG cases were classified according to different clinical characteristics based on a complex network and an integrated clinical research platform, and the correlation analysis of disease - symptom - syndrome type - tongue and pulse - traditional Chinese medicine was conducted on the classification results, to help identify more accurate CAG disease subtypes to help clinical precision medicine of CAG. A preliminary network pharmacological analysis was performed to search for relevant molecular linkages.

Our study has several potential limitations. The first is the sample size. In the analysis, we included only 5,132 patients. In addition, this was a single-centre study. In future studies, more patients should be included, and multicentre studies should be conducted to ensure the richness of the results. Finally, this study only conducted a network pharmacological correlation analysis of symptoms and Chinese medicine in the three identified subgroups to explore the broad possible molecular links between symptoms and Chinese medicine. This study is mainly used as a preliminary basis. Next, we will improve on these limitations and conduct further animal and cytological experiments to try and verify the relevant molecular associations found in this study.

## Data Availability

The original contributions presented in the study are included in the article/Supplementary Material, further inquiries can be directed to the corresponding authors.
