# Supplementary material for: Treating chronic atrophic gastritis: identifying sub-population based on real-world TCM electronic medical records
Source: Front Pharmacol. 2024 Aug 7;15:1444733. doi: 10.3389/fphar.2024.1444733 (PMC11335612; doi:10.3389/fphar.2024.1444733)
Supplement: Supplementary file 4 [file Table7.DOCX]

| designation | Family | Source |
| --- | --- | --- |
| Scutellaria baicalensis | Lamiaceae | [World Checklist](https://powo.science.kew.org/about-wcvp) ( [High Taxonomic Confidence](https://mpns.science.kew.org/mpns-portal/faq#TaxConfLevel)) |
| Smilax glabra | Smilacaceae | [World Checklist](https://powo.science.kew.org/about-wcvp) ( [High Taxonomic Confidence](https://mpns.science.kew.org/mpns-portal/faq#TaxConfLevel)) |
| Picrorhiza kurroa | Plantaginaceae | [World Checklist](https://powo.science.kew.org/about-wcvp) ( [High Taxonomic Confidence](https://mpns.science.kew.org/mpns-portal/faq#TaxConfLevel)) |
| Lilium lancifolium | Liliaceae | [World Checklist](https://powo.science.kew.org/about-wcvp) ( [High Taxonomic Confidence](https://mpns.science.kew.org/mpns-portal/faq#TaxConfLevel)) |
| Artemisia scoparia | Asteraceae | [World Checklist](https://powo.science.kew.org/about-wcvp) ( [High Taxonomic Confidence](https://mpns.science.kew.org/mpns-portal/faq#TaxConfLevel)) |
| Smilax glabra | Smilacaceae | [World Checklist](https://powo.science.kew.org/about-wcvp) ( [High Taxonomic Confidence](https://mpns.science.kew.org/mpns-portal/faq#TaxConfLevel)) |
| Cyperus rotundus | Cyperaceae | [World Checklist](https://powo.science.kew.org/about-wcvp) ( [High Taxonomic Confidence](https://mpns.science.kew.org/mpns-portal/faq#TaxConfLevel)) |
| Angelica sinensis | Apiaceae | [World Checklist](https://powo.science.kew.org/about-wcvp) |
| Conioselinum anthriscoides 'Chuanxiong' | Apiaceae | [WorldChecklist](https://powo.science.kew.org/about-wcvp)( [Medium Taxonomic Confidence](https://mpns.science.kew.org/mpns-portal/faq#TaxConfLevel)) |
| Paeonia lactiflora | Paeoniaceae | [World Checklist](https://powo.science.kew.org/about-wcvp) ( [High Taxonomic Confidence](https://mpns.science.kew.org/mpns-portal/faq#TaxConfLevel)) |
| Citrus medica | Rutaceae | [World Checklist](https://powo.science.kew.org/about-wcvp)([Medium Taxonomic Confidence)](https://mpns.science.kew.org/mpns-portal/faq#TaxConfLevel) |
| Solanum nigrum | Solanaceae | [World Checklist](https://powo.science.kew.org/about-wcvp)([Medium Taxonomic Confidence)](https://mpns.science.kew.org/mpns-portal/faq#TaxConfLevel) |
| Cremastra appendiculata | Orchidaceae | [World Checklist](https://powo.science.kew.org/about-wcvp) ( [High Taxonomic Confidence](https://mpns.science.kew.org/mpns-portal/faq#TaxConfLevel)) |
| Quanxie | Buthidae | http://www.yaobw.cn/ |
| Jiangcan |  | http://www.yaobw.cn/ |

All plant species are taxonomically validated and presented in tabular form.(http://mpns.kew.org/mpns-portal/), scorpion and fried silkworm is verified in China Pharmaceutical Labeling website (http://www.yaobw.cn/).
